# Oral health and social and emotional well-being in a birth cohort of Aboriginal Australian young adults

**DOI:** 10.1186/1471-2458-11-656

**Published:** 2011-08-19

**Authors:** Lisa M Jamieson, Yin C Paradies, Wendy Gunthorpe, Sheree J Cairney, Susan M Sayers

**Affiliations:** 1 Australian Research Center for Population Oral Health, The University of Adelaide, South Australia 5005, Australia; 2 McCaughey Centre and Onemda VicHealth Koori Health Unit, University of Melbourne, Victoria, Australia; 3 Menzies School of Health Research, Charles Darwin University, Darwin, Australia

## Abstract

**Background:**

Social and emotional well-being is an important component of overall health. In the Indigenous Australian context, risk indicators of poor social and emotional well-being include social determinants such as poor education, employment, income and housing as well as substance use, racial discrimination and cultural knowledge. This study sought to investigate associations between oral health-related factors and social and emotional well-being in a birth cohort of young Aboriginal adults residing in the northern region of Australia's Northern Territory.

**Methods:**

Data were collected on five validated domains of social and emotional well-being: anxiety, resilience, depression, suicide and overall mental health. Independent variables included socio-demographics, dental health behaviour, dental disease experience, oral health-related quality of life, substance use, racial discrimination and cultural knowledge.

**Results:**

After adjusting for other covariates, poor oral health-related items were associated with each of the social and emotional well-being domains. Specifically, anxiety was associated with being female, having one or more decayed teeth and racial discrimination. Resilience was associated with being male, having a job, owning a toothbrush, having one or more filled teeth and knowing a lot about Indigenous culture; while being female, having experienced dental pain in the past year, use of alcohol, use of marijuana and racial discrimination were associated with depression. Suicide was associated with being female, having experience of untreated dental decay and racial discrimination; while being female, having experience of dental disease in one or more teeth, being dissatisfied about dental appearance and racial discrimination were associated with poor mental health.

**Conclusion:**

The results suggest there may be value in including oral health-related initiatives when exploring the role of physical conditions on Indigenous social and emotional well-being.

## Background

In the Indigenous Australian context, social and emotional well-being is a broad concept reflecting a holistic understanding of life and health. It includes mental health, as well as cultural, spiritual and social well-being [1]. The concept also attempts to encompass the impact of grief and trauma through colonisation, separation from families, and loss of land and culture on Indigenous Australians [2]. The social and emotional well-being of many Indigenous Australians is poor, for example, in the 2004-05 National Aboriginal and Torres Strait Islander Health survey, Indigenous Australians were twice as likely to report high or very high levels of psychological distress as non-Indigenous Australians. The proportion was higher among Indigenous females compared with Indigenous males [3]. Indigenous Australians were also more than twice as likely to be hospitalised for intentional self-harm as non-Indigenous people [3].

A range of factors have been described as contributing to poor social and emotional well-being among Indigenous Australians. These include social determinants of health such as poor education, employment, income and housing as well as racism and level of cultural knowledge [1]. While there is some evidence that infection has a direct impact on mental ill health [4], in general there is limited documentation of the role of physical conditions on Indigenous social and emotional well-being. There is even less documentation on associations with oral health-related conditions.

Oral health is an integral component of overall health and well-being. Oral diseases are not only major causes of infection and tooth loss; but may cause debilitating pain and difficulties with eating/speaking, as well as limit social interactions [5-7]. The impact of oral disease is not confined to the mouth, with well established associations between chronic oral infections and heart [8] and lung diseases, diabetes [9], stroke [10] and pre-term low birth weight [11,12].

In Australia, the oral health needs of the Indigenous population are equivalent to those in nations such as Pakistan and Uzbekistan [13]. There are many reasons why preventable oral diseases are highly prevalent among this group. Financial barriers are frequently cited, together with lack of access to service providers due to social determinants of health [14]. Poor oral health has a substantial impact on quality of life [15], with poor oral health-related quality of life, in turn, having a substantial impact on daily performance and general life satisfaction [16].

According to the literature, there are three oral health-related domains that have theoretically plausible associations with social and emotional well-being. These include *behaviours *such as organisation of appropriate dental care (associated with confidence and coping) [17] and regular toothbrushing (associated with self-esteem) [18], *dental disease experience *such as presence of untreated dental decay [19] and *oral health-related quality of life*, such as toothache causing sleep dysregulation [20] and dissatisfaction with dental appearance [21].

We set out to assess whether (i) there were associations between oral health-related outcomes and social and emotional well-being and (ii) if yes, whether associations between oral health-related outcomes and social and emotional well-being persisted after adjusting for socio-demographic, substance use, racial discrimination and cultural knowledge in a sample of Indigenous Australian young adults.

## Methods

### Data source

Data from Wave-3 of the Aboriginal Birth Cohort (ABC) study, a prospective, longitudinal investigation of a birth cohort of Indigenous Australians was utilised. Babies were eligible for enrolment if they were live born singletons delivered at the Royal Darwin Hospital, Northern Territory, between January 1987 and March 1990 to a mother recorded as Aboriginal [22]. Within the local region at that time, 90 percent of pregnant Aboriginal mothers come to the Royal Darwin Hospital to deliver their babies [23].

Follow-ups were done at mean ages 4, 11 and most recently 18 years (Wave-3), when participants were located in more than 40 communities across the Northern Territory's Top End. The Human Research Ethics Committee of the Northern Territory Department of Health and Community Services and Menzies School of Health Research (including an Aboriginal sub-committee with absolute right of veto) granted ethics approval for each assessment phase. Study members gave informed consent before participating. Data are not openly available.

### Social and emotional well-being

Participants completed 'Strong Souls'; a validated and reliable tool for screening social and emotional well-being among Indigenous young adults in the Northern Territory [24]. The instrument included constructs pertaining to anxiety, resilience, depression, suicide risk and overall mental health; derived through factor analysis. Participants were asked in an interview how often they felt or experienced symptoms in the past few months, with response options including 'not much' (0), 'little bit' (1), 'fair bit' (2) and 'lots' (3). Scale scores were computed by simple item summation. A low score ('not much') indicated minimal or no issues and a high score ('fair bit' or 'lots') indicated substantial mental health issues. For resilience items, a low score indicated disagreement with the statement, indicative of lower levels of resilience and vice versa.

Anxiety comprised the following six items (high scores indicate high anxiety; possible range 0-18):

● Have you felt so sad that nothing could cheer you up?

● Have you felt so worried you start to shake?

● Have you felt so worried it was hard to breathe?

● Have you felt so worried you got really sweaty?

● Have you felt so worried you got sick in the guts?

● Have you felt so worried you got dizzy?

Resilience comprised the following nine items (high scores indicate high resilience; possible range 0-27):

● You have a strong family who help each other?

● You know lots about whitefella ways?

● You know someone who is a really good person?

● You laugh and make jokes a lot?

● You are really into something like music, cars, clothing etc?

● You are a good son or daughter to your family?

● You got an older person looking out for you?

● You got lots of friends?

● When you are upset you can usually talk to someone about it?

Depression comprised the following seven items (high scores indicate high depression; possible range 0-21):

● Have trouble sleeping?

● Get angry or wild real quick?

● Hard to focus/thinking all over the place?

● Had too many bad moods?

● Felt pretty lonely much of the time?

● Got angry or wild and stayed that way for a long time?

● Felt like giving up- no point in trying?

Suicide risk comprised the following three items (high scores indicate high suicide risk; possible range 0-9):

● Have you wished you were dead?

● Felt like hurting yourself?

● Have you felt like killing yourself?

Overall mental health was calculated by summing scores for anxiety, depression and suicide risk. Higher scores were indicative of poorer mental health, with the possible range of scores being 0 to 48. Participants identified as being at risk of poor mental health were referred to the local mental health service. An immediate consultation was arranged with mental health professionals for participants who were identified as being at risk of suicide or self harm. Aboriginal mental health workers and traditional healers were also made available to at-risk participants through their local health centre.

### Oral health-related risk indicators

Participants were invited to take part in a dental examination and to complete a self-report dental questionnaire [25]. Based on the literature, three domains of oral health-related outcomes were used to calculate associations with social and emotional well being: dental behaviours, dental disease experience and oral health-related quality of life.

#### Dental behaviour

Risk indicators relating to dental behaviours were defined as 'having seen a dentist before', 'age when last saw dentist', 'dental fear', 'toothbrush ownership', 'toothbrushing frequency' and 'age of toothbrushing onset.'

#### Dental disease experience

Information about clinical oral health status was collected during standardised clinical examinations conducted by two dentists who had been clinically calibrated against the gold standard examiner for the National Survey of Adult Oral Health. Examining dentists followed a standardised protocol to record levels of tooth loss, dental decay experience and periodontal disease. The DMFT (sum of decayed, missing and filled teeth in the permanent dentition) index was used to assess dental caries outcomes. All teeth present were divided into five tooth surfaces; occlusal/incisal, mesial, buccal, palatal/lingual and distal. Each dental surface was assessed and categorised using visual criteria only. Untreated dental decay (DT) was defined as 'cavitation of enamel or dentinal involvement or both being present' or 'visible caries that is contiguous with a restoration'. Filled due to decay (FT) was recorded when a tooth contained one or more permanent restorations placed to treat caries, while missing was recorded when a tooth had been extracted due to pathology (MT). Possible ranges of scores for DMFT, DT and FT are 0 to 32 for each respective measure. Dental disease experience measures were considered as percent DT > 0, percent FT > 0, percent DMFT > 0 and mean DMFT. **Participants who required dental care were provided with a referral form to the nearest public health dental service provider detailing the treatment required**.

The US Centres for Disease Control and Prevention and the American Academy of Periodontology definitions were used to describe moderate and severe periodontal disease; whereby moderate periodontal disease was defined as the presence of either two sites between adjacent teeth with 4 mm+ attachment loss, or at least two such sites with 5 mm+ pockets. Severe periodontal disease was classified as having at least two sites between adjacent teeth with 6 mm+ attachment loss and with at least one 5 mm+ pocket [26].

Repeat examinations to assess examiner reliability were not possible due to logistical and time constraints imposed by the study's multidisciplinary nature.

#### Oral health-related quality of life

Risk indicators that encapsulated oral health-related quality of life included 'frequency of dental pain', 'removal of tooth or teeth because of pain' and 'satisfaction with dental appearance'.

### Covariates

Covariates included socio-demographic, substance use, racial discrimination and cultural knowledge items.

#### Socio-demographic

Sex, residential location, source of household income (household income itself was not collected), household size and car ownership were included. Location was dichotomised into 'regional', which included participants living in the three regional centres, and 'rural/remote' which included participants living outside the regional jurisdictions. Source of household income was defined as 'job' (i.e. employment) or 'welfare' (i.e. unemployment or various government welfare programs). Household size was assessed by the question 'How many people stayed in your house last night?' while car ownership was measured by the question 'Does someone in your house own a car?' Household size was dichotomised into response options of 'four or less' and 'five or more'.

#### Substance use

As part of the social and emotional well-being questionnaire, participants were asked 'how much petrol/marijuana/tobacco/alcohol do you sniff/smoke/drink' with response options including (1) 'Never or only tried it once', (2) 'Used to sniff/smoke/drink, but not any more' and (3) 'Still sniff/smoke/drink sometimes'. For purposes of this analysis the latter two response options were combined.

#### Discrimination

Based on items used in the National Aboriginal and Torres Strait Islander Health Survey [3], respondents were asked if they had ever been treated unfairly or discriminated against because they were Aboriginal, with response options dichotomised into 'not really' and 'little bit, fair bit and lots of times'.

#### Cultural knowledge

Participants were asked if they knew a lot about their Aboriginal culture. Responses were dichotomised into 'lots' and 'fair bit, little bit and not much'.

### Data analytic approach

Univariate distributions of the five social and emotional well-being domains were determined. ANOVA was used to test the bivariate relationship between the five social and emotional well-being domains with socio-demographic, behavioural, dental disease experience, oral health impact, substance use, discrimination and cultural knowledge factors. Correlation tests between the independent variables confirmed the existence of weak associations between items in a given group (Pearson's correlation coefficient range 0.1-0.4). Based on the criteria that Pearson's correlation coefficients need to be 0.8+ to be excluded due to multicollinearity [27], no items were excluded.

To determine associations between the oral health-related factors and the five social and emotional well-being constructs, explanatory variables were entered into a series of five linear regression models, one for each of the social and emotional well-being constructs. Initial models were constructed containing all independent variables significantly associated with the outcome variables in bivariate analysis. The final regression models were constructed by removing covariates that were not statistically significant at the p < 0.05 level. Independent variables were classified into socio-demographic, dental behaviour, dental disease experience, oral health impact, substance use, discrimination and cultural knowledge items. Residual plots (plots of the standardized residuals as a function of standardized predicted values) of the models were examined to check normality assumptions, with the key properties of the residuals being lack of correlation with the independent variables, independence and homoscedasticity [28]. Because these properties were observed within the dataset for the variables of interest, the use of linear regression was justified. Data were analysed using SPSS version 15.

## Results

Complete data were available for 336 participants. In bivariate analyses, females had higher levels of anxiety, depression, suicide ideation and overall poor mental health (Table 1). Males, those with a job, those with a household size of 4 or less people the previous night and car owners had higher levels of resilience. Participants reporting late onset of toothbrushing had higher levels of anxiety, while those who had visited a dentist before or who owned a toothbrush had higher levels of resilience (Table 2).

**Table 1:** Social and emotional well-being of ABC study participants by socio-demographic factors

	Anxiety mean (95% CI)	Resilience mean (95% CI)	Depression mean (95% CI)	Suicide mean (95% CI)	Mental health mean (95% CI)
**Total**	2.07 (1.78-2.36)	21.64 (21.19-22.09)	4.06 (3.71-4.41)	0.52 (0.38-0.66)	6.65 (6.04-7.26)
**Range**	0-18	5-27	0-17	0-9	0-30
					
**Socio-demographic**					
Sex					
Male	1.54 (1.21-1.87)*	22.74 (22.13-23.35)*	3.34 (2.85-3.83)*	0.24 (0.14-0.34)*	5.12 (4.39-5.85)*
Female	2.53 (2.08-2.98)	20.68 (20.05-21.31)	4.69 (4.22-5.16)	0.77 (0.55-0.99)	7.99 (7.11-8.87)
					
Residential location					
Regional	1.97 (1.38-2.56)	21.53 (20.57-22.49)	3.82 (3.08-4.56)	0.57 (0.26-0.88)	6.36 (5.11-7.61)
Rural/remote	2.10 (1.77-2.43)	21.67 (21.16-22.18)	4.13 (3.74-4.52)	0.51 (0.37-0.65)	6.74 (6.05-7.43)
					
Source of household income					
Job	1.39 (0.68-2.10)	23.52 (22.48-24.56)*	4.11 (3.03-5.19)	0.65 (0.16-1.14)	6.15 (4.31-7.99)
Welfare	2.18 (1.87-2.49)	21.34 (20.85-21.83)	4.06 (3.69-4.43)	0.50 (0.38-0.62)	6.73 (6.10-7.36)
					
Household size					
Four or less people	2.09 (1.48-2.70)	22.84 (21.96-23.72)*	4.31 (3.57-5.05)	0.61 (0.26-0.96)	7.01 (5.66-8.36)
Five or more people	2.09 (1.74-2.44)	21.28 (20.75-21.81)	3.94 (3.55-4.33)	0.49 (0.35-0.63)	6.51 (5.82-7.20)
					
Do you own a car?					
No	2.15 (1.84-2.46)	21.43 (20.94-21.92)*	4.09 (3.72-4.46)	0.55 (0.41-0.69)	6.79 (6.14-7.44)
Yes	1.36 (0.85-1.87)	23.58 (22.42-24.74)	3.79 (2.89-4.69)	0.27 (0.02-0.52)	5.42 (4.26-6.58)

*P < 0.05

**Table 2:** Social and emotional well-being of ABC study participants by behavioural factors

	Anxiety mean (95% CI)	Resilience mean (95% CI)	Depression mean (95% CI)	Suicide mean (95% CI)	Mental health mean (95% CI)
**Total**	2.07 (1.78-2.36)	21.64 (21.19-22.09)	4.06 (3.71-4.41)	0.52 (0.38-0.66)	6.65 (6.04-7.26)
**Range**	0-18	5-27	0-17	0-9	0-30
					
Have seen dentist before?					
No	2.06 (0.83-3.29)	19.56 (17.21-21.91)*	3.89 (2.62-5.16)	0.78 (0.27-1.29)	6.72 (4.78-8.66)
Yes	2.07 (1.78-2.36)	21.75 (21.30-22.20)	4.07 (3.72-4.42)	0.51 (0.37-0.65)	6.65 (6.02-7.28)
					
Age when last saw dentist					
12 years or less	2.12 (1.71-2.53)	21.53 (20.92-22.14)	4.01 (3.58-4.44)	0.46 (0.30-0.62)	6.58 (5.78-7.38)
13 years or more	1.98 (1.57-2.39)	22.15 (21.46-22.84)	4.15 (3.54-4.76)	0.59 (0.35-0.83)	6.72 (5.72-7.72)
					
Would you feel scared about going to the dentist?					
No	1.78 (1.43-2.13)	21.99 (21.28-22.70)	3.92 (3.35-4.49)	0.49 (0.29-0.69)	6.18 (5.32-7.04)
Little bit, fair bit, heaps	2.27 (1.84-2.70)	21.39 (20.80-21.98)	4.16 (3.73-4.59)	0.55 (0.39-0.71)	6.98 (6.16-7.80)
					
Toothbrush ownership					
No	2.28 (1.65-2.91)	20.71 (19.81-21.61)*	3.91 (3.30-4.52)	0.44 (0.22-0.66)	6.63 (5.43-7.83)
Yes	1.98 (1.67-2.29)	22.00 (21.49-22.51)	4.12 (3.71-4.53)	0.56 (0.40-0.72)	6.66 (5.97-7.35)
					
If yes, did brush teeth yesterday?					
No	1.69 (1.22-2.16)	21.96 (21.02-22.90)	3.62 (2.91-4.33)	0.32 (0.14-0.50)	5.63 (4.57-6.69)
Yes	2.10 (1.71-2.49)	22.10 (21.49-22.71)	4.34 (3.83-4.85)	0.65 (0.45-0.85)	7.08 (6.22-7.94)
					
If yes, what age when started to brush?					
When had little teeth	1.66 (1.29-2.03)*	22.47 (21.82-23.12)	4.21 (3.66-4.76)	0.47 (0.31-0.63)	6.34 (5.54-7.14)
When had big teeth	2.56 (1.99-3.13)	21.45 (20.53-22.37)	4.01 (3.34-4.68)	0.71 (0.38-1.04)	7.29 (6.00-8.58)

*P < 0.05

Those with one or more teeth with untreated dental decay, those with experience of one or more decayed, missing or filled teeth, those who had experienced dental pain in the last year or who were dissatisfied with dental appearance had higher mean levels of anxiety (Table 3). Experience of filled teeth was associated with resilience. Participants with experience of decayed, missing or filled teeth or who had experienced dental pain had higher levels of depression. Experience of decayed, missing or filled teeth and dissatisfaction of dental appearance was also associated with suicide risk. Those with untreated dental disease, those with one or more teeth that was decayed, missing or filled, who had experienced dental pain and who were dissatisfied with their dental appearance had poorer overall levels of mental health.

**Table 3:** Social and emotional well-being of ABC study participants by dental disease experience and oral health impact factors

	Anxiety mean (95% CI)	Resilience mean (95% CI)	Depression mean (95% CI)	Suicide mean (95% CI)	Mental health mean (95% CI)
**Total**	2.07 (1.78-2.36)	21.64 (21.19-22.09)	4.06 (3.71-4.41)	0.52 (0.38-0.66)	6.65 (6.04-7.26)
**Range**	0-18	5-27	0-17	0-9	0-30
					
**Dental disease experience**					
DT > 0					
No	1.45 (1.06-1.84)*	21.36 (20.34-22.38)	3.68 (3.07-4.29)	0.41 (0.21-0.61)	5.54 (4.62-6.46)*
Yes	2.30 (1.93-2.67)	21.74 (21.25-22.23)	4.20 (3.79-4.61)	0.57 (0.41-0.73)	7.07 (6.33-7.81)
FT > 0					
No	2.19 (1.86-2.52)	21.28 (20.75-21.81)*	3.93 (3.56-4.30)	0.50 (0.36-0.64)	6.63 (5.94-7.32)
Yes	1.58 (1.05-2.11)	23.00 (22.20-23.80)	4.58 (3.72-5.44)	0.61 (0.28-0.94)	6.77 (5.46-8.08)
DMFT > 0					
No	1.32 (0.95-1.69)*	21.11 (19.95-22.27)	3.25 (2.68-3.82)*	0.36 (0.16-0.56)*	4.92 (4.04-5.80)*
Yes	2.29 (1.94-2.64)	21.79 (21.30-22.28)	4.30 (3.89-4.71)	0.57 (0.41-0.73)	7.16 (6.43-7.89)
					
Mean DT (Pearson's correlation coefficient)	0.14*	-0.02	0.05	0.15*	0.13*
					
Moderate or severe periodontal disease					
No	2.09 (1.74-2.44)	21.78 (21.25-22.31)	4.07 (3.68-4.46)	0.58 (0.42-0.74)	6.74 (6.03-7.45)
Yes	2.01 (1.48-2.54)	21.25 (20.37-22.13)	4.03 (3.34-4.72)	0.37 (0.19-0.55)	6.42 (5.30-7.54)
					
**Oral health impact**					
How have your teeth been since last wet?					
All good, none hurting	1.62 (1.29-1.95)*	21.85 (21.24-22.46)	3.72 (3.27-4.17)*	0.43 (0.27-0.59)	5.76 (5.05-6.47)*
Some good, some hurting or none good, all hurting	2.64 (2.15-3.13)	21.36 (20.67-22.05)	4.50 (3.97-5.03)	0.65 (0.43-0.87)	7.79 (6.79-8.79)
					
Have you ever had a tooth removed because it hurt too much?					
No	1.96 (1.65-2.27)	21.75 (21.24-22.26)	3.93 (3.56-4.30)	0.47 (0.33-0.61)	6.35 (5.70-7.00)*
Yes	2.52 (1.87-3.17)	21.18 (20.20-22.16)	4.61 (3.73-5.49)	0.73 (0.40-1.06)	7.87 (6.42-9.32)
					
Do you think your teeth look ok?					
All good	1.52 (1.17-1.87)*	22.18 (21.45-22.91)	3.67 (3.06-4.28)	0.30 (0.16-0.44)*	5.48 (4.60-6.36)*
Some good or none good	2.38 (1.99-2.77)	21.34 (20.77-21.91)	4.28 (3.87-4.69)	0.65 (0.47-0.83)	7.31 (6.53-8.09)

*P < 0.05

Marijuana use was associated with depression (Table 4), while alcohol consumption demonstrated inconsistent associations; greater resilience, lower anxiety but higher depression. Experiencing discrimination was associated with anxiety, depression, suicide risk and overall poor mental health, making it the only factor related to the four negative social and emotional well-being outcomes. Participants who knew a lot about their Aboriginal culture reported higher resilience.

**Table 4:** Social and emotional well-being of ABC study participants by substance use, discrimination and cultural knowledge factors

	Anxiety mean (95% CI)	Resilience mean (95% CI)	Depression mean (95% CI)	Suicide mean (95% CI)	Mental health mean (95% CI)
**Total**	2.07 (1.78-2.36)	21.64 (21.19-22.09)	4.06 (3.71-4.41)	0.52 (0.38-0.66)	6.65 (6.04-7.26)
**Range**	0-18	5-27	0-17	0-9	0-30
					
**Substance use**					
Tobacco					
No	2.16 (1.63-2.69)	21.69 (20.85-22.53)	3.98 (3.25-4.71)	0.53 (0.33-0.73)	6.67 (5.49-7.85)
Yes	2.03 (1.68-2.38)	21.62 (21.07-22.17)	4.10 (3.71-4.49)	0.52 (0.36-0.68)	6.65 (5.94-7.36)
					
Marijuana					
No	2.01 (1.68-2.34)	21.50 (20.93-22.07)	3.76 (3.33-4.19)*	0.49 (0.33-0.65)	6.26 (5.52-7.00)
Yes	2.16 (1.63-2.69)	21.85 (21.11-22.59)	4.53 (3.96-5.10)	0.58 (0.36-0.80)	7.27 (6.25-8.29)
					
Alcohol					
No	2.37 (1.94-2.80)*	20.95 (20.32-21.58)*	3.75 (3.55-3.95)*	0.57 (0.37-0.77)	6.69 (5.87-7.51)
Yes	1.71 (1.34-2.08)	22.46 (21.83-23.09)	4.43 (4.25-4.61)	0.47 (0.29-0.65)	6.61 (5.73-7.49)
					
**Discrimination**					
Treated unfairly or discriminated against because are Aboriginal?					
Not really	1.66 (1.37-1.95)*	21.75 (21.20-22.30)	3.62 (3.50-3.74)*	0.40 (0.28-0.52)*	5.68 (5.01-6.35)*
Little bit, fair bit, lots	2.93 (2.32-3.54)	21.40 (20.62-22.18)	4.98 (4.69-5.27)	0.78 (0.49-1.07)	8.69 (7.53-9.85)
					
**Cultural knowledge**					
Do you know a lot about your Aboriginal culture?					
Not much, little bit, fair bit	1.90 (1.49-2.31)	20.49 (19.65-21.33)*	4.18 (3.59-4.77)	0.52 (-0.50-1.54)	6.61 (5.67-7.55)
Lots	2.16 (1.77-2.55)	22.27 (21.76-22.78)	4.00 (3.57-4.43)	0.53 (-0.51-1.57)	6.68 (5.92-7.44)

*P < 0.05

In multivariate modeling, anxiety was associated with being female, having one or more decayed teeth and having experienced discrimination, while being male, having a job, owning a toothbrush, having one or more filled teeth and knowing a lot about Aboriginal culture were associated with resilience (Table 5). Depression was associated with being female, having experienced dental pain in the past year, use of marijuana, use of alcohol and having felt discriminated against, while being female, having experience of untreated dental decay and discrimination were associated with suicide risk. Overall poor mental health was associated with being female, having experience of dental disease in one or more teeth, being dissatisfied about dental appearance and having experienced discrimination. In multivariate models, being female was associated with the range of social and emotional well-being outcomes as were experiences of discrimination. Other associations found in bivariate analyses were no longer common to multiple outcomes in multivariate models. Alcohol no longer acted as a protective indicator in these models but remained associated with depression.

**Table 5:** Multivariate models of ABC study participants with experience of poor social and emotional well-being

	Anxiety	Resilience	Depression	Suicide	Mental health
	B (95% CI)	B (95% CI)	B (95% CI)	B (95% CI)	B (95% CI)
**Socio-demographic**					
Sex					
Male	ref	ref	ref	ref	ref
Female	0.88 (0.32-1.44)	-1.95 (-2.81--1.09)	1.51 (0.84-2.18)	0.49 (0.25-0.74)	2.47 (1.33-3.60)
Source of household income					
Job	-	ref	-	-	-
Welfare	-	-1.93 (-3.17-0.70)	-	-	-
					
**Dental behavioural**					
Toothbrush ownership					
No	-	ref	-	-	-
Yes	-	1.10 (0.16-2.04)	-	-	-
**Dental disease experience**					
DT > 0					
No	ref	-	-	-	-
Yes	0.78 (0.16-1.40)	-	-	-	-
FT > 0					
No	-	ref	-	-	
Yes	-	1.71 (0.66-2.77)	-	-	
DMFT > 0					ref
No	-	-	-	-	1.38 (0.04-2.80)
Yes	-	-	-	-	
Mean DT	-	-	-	0.034 (0.01-0.06)	-
					
**Oral health impact**					
How have your teeth been since last wet?					
All good, none hurting	-	-	ref	-	-
Some good, some hurting or none good, all hurting	-	-	0.74 (0.08-1.40)	-	-
Do you think your teeth look ok?					
All good	-	-	-	-	ref
Some good or none good	-	-	-	-	1.23 (0.01-2.47)
					
**Substance use**					
Marijuana					
No	-	-	ref	-	-
Yes	-	-	0.78 (0.07-1.49)	-	-
Alcohol	-	-		-	-
No	-	-	ref	-	-
Yes			0.91 (0.21-1.62)		
					
**Racial Discrimination**					
Treated unfairly or discriminated against because you are Aboriginal?					
Not really	ref	-	ref	ref	ref
Little bit, fair bit, lots	1.18 (0.59-1.78)	-	1.37 (0.67-2.07)	0.34 (0.08-0.60)	2.79 (1.59-3.99)
**Cultural knowledge**					
Do you know a lot about your Aboriginal culture?	-		-	-	-
Not much, little bit, fair bit	-	ref	-	-	-
Lots		1.55 (0.66-2.44)			

## Discussion

Our findings indicate that poor oral health-related outcomes - as assessed by dental behaviours, dental disease experience and oral health-related quality of life - were associated with poor social and emotional well-being - as assessed by anxiety, resilience, depression, suicide risk and mental health - in a birth cohort of Indigenous Australian young adults, after adjustment for socio-demographics, substance use, discrimination and cultural knowledge. The results highlight the importance of including oral health-related factors when determining the broad range of factors associated with poor Indigenous social and emotional well-being, and support inclusion of oral health-related initiatives when developing interventions aimed at fostering social and emotional development among Indigenous Australian groups.

It is important to bear in mind that, being a longstanding birth cohort, the study was not designed at the outset to examine the relationship between social and emotional wellbeing and oral health. The sample size was determined at birth and not able to be increased in later stages. This study represents the largest cohort of an Australian Indigenous population that has ever been assembled, with this, in turn, being the largest study to examine social and emotional wellbeing and oral health in an Indigenous young adult population.

Before discussing implications of the findings, it is important to consider the study's limitations. We have suggested that oral health-related factors are associated with poor social and emotional well-being, but it is not possible to determine the exact causal pathway. The true relationship may be bi-directional. Although the study is longitudinal in design, oral health and social and emotional well-being items were only collected in the most recent phase. The self-report nature of many of the items may have led to an under-estimation of these factors. Although high concordance between self-report and biological measures of factors such as smoking have been reported [29], this has not been explored among a marginalised group such as Indigenous Australians, where incorrect responses may be given for any number of reasons (social desirability bias, difficulty understanding English, recall and yea-saying bias).

It is worth expanding upon each of the oral health-related components that were associated with social and emotional well-being in this study.

### Dental behaviour

The only dental behaviour significantly associated with social and emotional well-being in multivariate analysis was toothbrush ownership, with ownership of a toothbrush being positively associated with resilience. In Kuwait, Honkala and colleagues [18] reported associations between recommended brushing and life- or school-satisfaction and self-esteem indicators. Factors additionally associated with resilience in our study included being male, having a job, having a filling and having more cultural knowledge. Employment and cultural identification are recognised as being among the broader determinants of health associated with social and emotional well-being [30,31].

### Dental disease experience

Untreated dental disease in our study was associated with anxiety. The most intuitive reason for this is because untreated dental disease can cause pain, limit function and look unsightly [19]. Anxiety was also associated with being female and discrimination. In the literature it is reported that females are more likely to feel concerned about the appearance of their teeth [32]. Facial attractiveness has also been found to affect social attitudes and actions, and is important in employment situations [33,34].

Experience of one or more decayed, missing or filled teeth was associated with poor mental health. Most evidence in the literature suggests the opposite; that poor mental health is a risk indicator for dental disease. Severity of untreated dental disease was associated with suicide risk in this study. There is limited evidence of this relationship in the literature, although at a qualitative level, suicide has been linked to toothache in the Indigenous Australian context [35]. The most common cause of toothache is severe, untreated dental disease [5].

### Oral health-related quality of life

In our study, experience of dental pain in the last year was associated with depression. Depression is a complex psychological condition with many elements of pain acting as an underlying stimulus [36]. It has been reported that pain and depression share underlying neurochemical mechanisms [36], with stressful life events preceeding the onset of symptoms of both chronic facial pain [37] and depression [38]. It is quite possible that they may have a common underlying pathophysiology, with evidence suggesting that a larger proportion of chronic pain patients may develop depression than patients with various other chronic medical conditions [39].

Dissatisfaction with dental appearance was associated with poor overall mental health. A study of young adults in Japan found that dental malocclusion had a negative impact on self-reported mental health status [21] and, among an adult population in Karachi, perceived severity of malocclusion was significantly associated with poor psychosocial well-being [40]. People suffering from problems in their tempero-mandibular joints (the joint linking the lower jaw with the rest of the skull) are more likely to suffer from mental disorders [41] and to be more severely depressed than healthy individuals [42]. Indeed, depression has a high co-morbidity with chronic facial pain [43]. In a qualitative study of rural-dwelling Indigenous Australian adults, it was reported that *'...It affects your whole body, having toothache, your way of thinking...' *and '..*You see a lot of young people today with missing teeth and stuff....some feel shame about it too you know, like how it looks and everything.' *[35].

Additional associations with poor social and emotional well-being among Indigenous young adults in this study warrant mention. Others have recognised that Indigenous Australian females (as with Australian females in general) have poorer social and emotional well-being than their male counterparts [3]. Although reasons for this disparity are unclear, they may include Indigenous females having greater social responsibilities, less career or education options, early motherhood and general chronic disease comorbidity [3]. Having a job was associated with resilience, which is intuitive given that maintaining any form of employment requires some degree of commitment and responsibility [44]. Substance misuse, in the form of alcohol and marijuana, were associated with depression, supported in the literature for both Indigenous [45] and non-Indigenous [46] populations. Discrimination was associated with four of the five social and emotional well-being domains; anxiety, depression, suicide risk and overall mental ill health. This is an important finding that mirrors emerging Australian [47-51] and international evidence that racism is an important cause of ill-health [52,53]; suggesting that further investigation of this topic in Australia is warranted [54]. Cultural knowledge was associated with resilience, which is again supported by the literature.

It is important to consider the relevance of the findings for dental health policy and practice, specifically in delivering services to people in locations in which this study was undertaken. Indigenous young adults in the Northern Territory who own a means-tested health care card are eligible for free dental services through the public sector. Although these services are available on a reasonably regular basis, our findings indicate that many social and emotional-related factors may contribute to an individual not presenting for recommended care. Most Indigenous Australians visit a dentist because of a problem [55]. Increased understanding of the associations between social and emotional well-being and oral health may help encourage policies that reflect an appreciation of this, which may, in time, result in more preventive-based dental visiting patterns.

## Conclusion

In summary, our findings show that after adjustment for important socio-demographic, substance use, discrimination and cultural knowledge, poor oral health-related outcomes were associated with poor social and emotional well-being in a birth cohort of Indigenous Australian young adults. The results support the supposition that social and emotional well-being is a complex condition associated with many factors, including oral health-related outcomes. We were unable to determine true causal pathways between the oral health and social and emotional wellbeing findings, but believe the findings are still useful, particularly in providing a more comprehensive understanding of holistic health parameters. Clearly, further research is required to elucidate more clearly the causal pathways between these outcomes.

## Competing interests

The authors declare that they have no competing interests.

## Authors' contributions

LMJ, WG and SMS participated in data collection and manuscript preparation. YCP and SJC participated in data management and manuscript preparation. All authors were involved in revising the manuscript for important intellectual content and read and approved the final manuscript.

## Pre-publication history

The pre-publication history for this paper can be accessed here:

http://www.biomedcentral.com/1471-2458/11/656/prepub
